# Diagnosis of acromegaly: black, white… and sometimes gray!

**DOI:** 10.1590/2359-3997000000231

**Published:** 2016-11-01

**Authors:** Philippe Chanson

**Affiliations:** 1 Hôpitaux Universitaires Paris-Sud Le Kremlin Bicêtre France Assistance Publique-Hôpitaux de Paris (P.C.), Hôpitaux Universitaires Paris-Sud, Hôpital de Bicêtre, Service d’Endocrinologie et des Maladies de la Reproduction, Le Kremlin Bicêtre, France; 2 Fac Med Paris Sud Univ Paris-Sud France Inserm 1185, Fac Med Paris Sud, Univ Paris-Sud,; Université Paris-Saclay Le Kremlin-Bicêtre France Université Paris-Saclay, Le Kremlin-Bicêtre, France

Disease definitions often rely on cutoff values chosen to help distinguish a pathological condition from a healthy state. This is particularly true in endocrinology, where hormone hypersecretion or hyposecretion needs to be distinguished from physiological secretion. In general, endocrinological disease states are associated with clearly pathological hormone levels, largely above or below the proposed diagnostic cutoff. In acromegaly for example (1,2), most patients have obvious clinical signs and IGF-I levels markedly above the upper normal limit (ULN). But how is the ULN determined, and what does it signify? In general, the normal range of a biological marker is based on values observed in the healthy general population. If values follow a Gaussian distribution (with as many values above as below the mean), the ULN is generally set at the 97.5^th^ percentile, corresponding more or less to the mean + 2 standard deviations (SD), while the lower limit of normal is the 2.5^th^ percentile, corresponding more or less to the mean - 2SD.

However, it is no simple matter to establish reference values for IGF-I. Indeed, serum IGF-I concentrations rise with age during childhood and puberty, while they fall with age in adults (3). Furthermore, the distribution of IGF-I values in an apparently healthy population is non Gaussian, necessitating the use of complex mathematical transformations to obtain reference intervals for a given age group. For this reason, it is crucial to generate reference values after stratifying a large healthy population into age groups (4). Another problem is that IGF-I concentrations are influenced by many factors other than the GH concentration, including nutritional status and BMI, the use of post-menopausal hormone replacement therapy and its route of administration (5-7), kidney and liver function, and diabetic status (8). Reference IGF-I values may therefore be influenced by the inclusion criteria used to select the reference population. Elsewhere, comparisons of IGF-I assay kits show that, even in the same healthy population, IGF-I reference ranges can differ: as a result, some individuals considered to have “high” IGF-I levels measured with one assay kit may have “normal” levels when another kit is used (9). Finally, by definition, 5% of the healthy population have IGF-I levels either above the 97.5^th^ percentile or below the 2.5^th^ percentile. This means that 2.5% of the normal healthy population may have IGF-I levels above the ULN. All these factors may explain why some of the subjects reported in the article by Rosario and Calsolari were found to have elevated IGF-I levels despite perfectly normal GH secretion (10). The fact that IGF-I levels were above the ULN not only at the first sampling but also at the subsequent measurement five years later suggests that IGF-I levels, like other biological parameters such as TSH, tend to be “set” at an individual level which varies very little, within a range narrower than that of the reference population (11).

GH levels are also clearly elevated in the vast majority of patients with acromegaly, both at baseline and in the oral glucose tolerance test (OGTT), making the biochemical diagnosis of acromegaly quite straightforward (2,12). However, it must be kept in mind that a few patients with clear clinical signs and high IGF-I levels may also have authentic acromegaly despite very low GH secretion, including a nadir of < 1 µg/l in the OGTT (13-18). These patients generally have a microadenoma, which can be difficult to visualize or may even have questionable pituitary MRI. Moreover, when GH output is low (basal level < 4 µg/l), the OGTT may sometimes be misleading, as GH levels can be suppressed below 0.3 µg/l in some patients with true acromegaly (17,19). The existence of these very rare cases means that all patients with clinical signs of acromegaly and elevated IGF-I levels should have the OGTT. If GH is suppressed to below 0.3 µg/l, acromegaly is unlikely but cannot be ruled out. As stated by Rosario and Calsolari, the most reasonable attitude is to monitor the patient and to repeat laboratory tests after a few months or years.

For the diagnosis of acromegaly, as in all fields of medicine, one must accept that not everything is black and white, and that there may be many shades of gray. As Osler put it, “*medicine is a science of uncertainty and an art of probability*” (20).
